# Early Life Inflammation and the Developing Hematopoietic and Immune Systems: The Cochlea as a Sensitive Indicator of Disruption

**DOI:** 10.3390/cells10123596

**Published:** 2021-12-20

**Authors:** Kelly S. Otsuka, Christopher Nielson, Matthew A. Firpo, Albert H. Park, Anna E. Beaudin

**Affiliations:** 1 Department of Pathology, University of Utah School of Medicine, Salt Lake City, UT 84112, USA; kelly.otsuka@path.utah.edu; 2 Division of Otolaryngology—Head and Neck Surgery, University of Utah School of Medicine, Salt Lake City, UT 84112, USA; nielson.chris@hsc.utah.edu (C.N.); albert.park@hsc.utah.edu (A.H.P.); 3 Department of Surgery, University of Utah, Salt Lake City, UT 84112, USA; matt.firpo@hsc.utah.edu; 4 Division of Hematology and Hematologic Malignancies, University of Utah School of Medicine, Salt Lake City, UT 84112, USA

**Keywords:** hematopoiesis, inflammation, cochlea, congenital infection, hematopoietic stem and progenitor cells, fetal-derived immune cells, cytomegalovirus, sensorineural hearing loss

## Abstract

Emerging evidence indicates that perinatal infection and inflammation can influence the developing immune system and may ultimately affect long-term health and disease outcomes in offspring by perturbing tissue and immune homeostasis. We posit that perinatal inflammation influences immune outcomes in offspring by perturbing (1) the development and function of fetal-derived immune cells that regulate tissue development and homeostasis, and (2) the establishment and function of developing hematopoietic stem cells (HSCs) that continually generate immune cells across the lifespan. To disentangle the complexities of these interlinked systems, we propose the cochlea as an ideal model tissue to investigate how perinatal infection affects immune, tissue, and stem cell development. The cochlea contains complex tissue architecture and a rich immune milieu that is established during early life. A wide range of congenital infections cause cochlea dysfunction and sensorineural hearing loss (SNHL), likely attributable to early life inflammation. Furthermore, we show that both immune cells and bone marrow hematopoietic progenitors can be simultaneously analyzed within neonatal cochlear samples. Future work investigating the pathogenesis of SNHL in the context of congenital infection will therefore provide critical information on how perinatal inflammation drives disease susceptibility in offspring.

It is now well recognized that immune dysfunction is a critical driver of pathology not only in many chronic diseases, including autoimmune diseases, cardiovascular disease, and neurodegenerative disease, but also in the origins of many acute diseases such as cancer [1]. Chronic inflammation, defined as a low-grade, sustained immune response in the absence of a specific inflammatory stimulus, eventually perturbs cellular and tissue function, and causes a breakdown in immune tolerance, ultimately leading to disease. The pathophysiological mechanisms that initially drive chronic inflammation and lead to disease pathogenesis, however, are poorly understood, and genetic risk factors alone cannot account for the incidence of most chronic diseases [2,3]. Defining the etiology of chronic diseases and understanding their pathogenesis are critical for implementing preventative measures and mitigating risk from disease.

The Developmental Origin of Health and Disease hypothesis (DOHaD), originally proposed by Barker, postulates that many chronic diseases can be traced to perturbations during early life [4,5]. The original hypothesis was based on epidemiological associations between poor maternal nutrition and low infant birthweight with later rates of cardiac disease and premature death in offspring [4,6]. This original finding has spawned decades of research in defining developmental origins of disease, which has since expanded to include the investigation of the effects of early life toxicant exposure, infection, and nutritional status, among other perturbations, on long-term health outcomes. The primary focus from a mechanistic angle has been on epigenetic modifications that potentially mediate long-term alterations to cellular and tissue function [7], particularly in the context of metabolic programming, but investigation is ongoing.

The immune system was virtually ignored in the context of investigating developmental origins of disease, due in part to the long-standing view that immune development was mostly linear in nature—as the organism matured, so did the immune system. The notion that most hematopoietic and immune cells were continuously replaced across the lifespan of the organism was also incongruent with the concept of early life programming. However, over the last decade or more, the advent of increasingly sophisticated genetic approaches, including fate mapping, single-cell sequencing, and clonal tracking, has challenged this perception. Studies using these approaches have further clarified that fetal hematopoiesis produces distinct immune cells [8,9,10] that persist into the postnatal period and contribute to adult immune function. Furthermore, accumulating evidence indicates that fetal-derived immune cells possess specialized functions and make unique contributions to tissue development [11,12,13,14], homeostasis [15], and immune function [16,17] that are distinct from function of immune cells produced during adulthood. Given this recent appreciation for the contribution of fetal immune development to adult immune function, we can now revisit the developmental origins of disease hypothesis from the lens of understanding how perinatal inflammation shapes disease susceptibility by affecting immune function from development onwards.

## 1. Perinatal Inflammation Shapes Offspring Immunity

**Figure 1:**
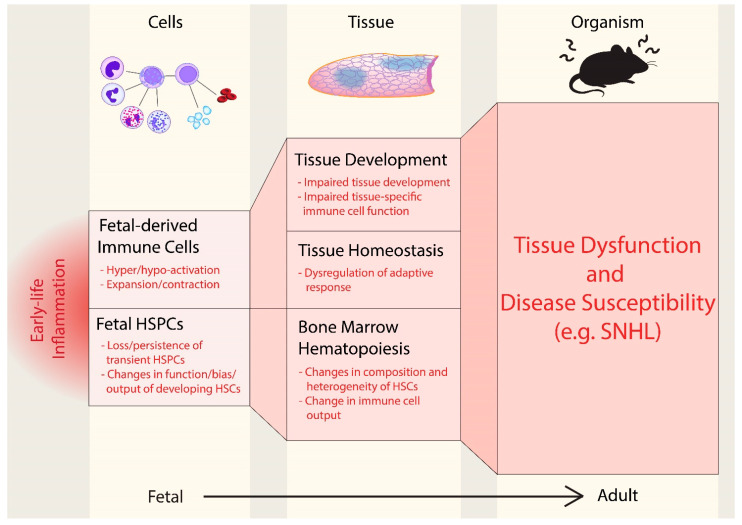
Early life inflammation as a driver of disease susceptibility. Perinatal inflammation can drive lasting changes to immune function from fetal development onwards by perturbing immune function at the cellular, tissue, and whole organism level. Impaired development of fetal-derived immune cells that influence tissue development and homeostasis can ultimately impair tissue function. Specifically, fetal-derived immune cell compartments can either be expanded or reduced by inflammation, or cells can by hyper- or hypo-activated. Perturbing the establishment and function of developing fetal hematopoietic stem and progenitor cells (HSPCs) by inducing loss or persistence of transient HSPCs, or shaping function and output of developing HSCs may also result in lasting changes to the composition or output of the adult hematopoietic stem cell (HSC) compartment. Ultimately, the effects of perinatal inflammation on both developing HSPCs and fetal-derived immune cells may reshape the trajectory of postnatal immunity and susceptibility to disease by impinging on tissue immunity and/or the output of the hematopoietic system.

The last three decades have witnessed a significant shift in understanding of how early microbe exposure influences lifelong health outcomes. Strachan proposed the highly influential “hygiene hypothesis” in 1989 [18], which encapsulated the idea that increased exposure to microbes in larger families was inversely correlated with a risk for asthma and atopy. We and others have recently reviewed the extent to which perinatal microbe exposure influences immune outcomes in offspring [19], including early response to vaccine [20], susceptibility to autoimmune and hyper-sensitivity diseases [21,22], and early response to infection [23]. The term “perinatal” encompasses both fetal as well early postnatal development, and captures not only continuity of development but also continuity of inflammation or exposure that occurs across this developmental window. Underlying these changes is the notion that the developing immune system must be “programmed” at some level by inflammation [19]. There is a significant gap, however, in understanding the mechanisms by which this programming occurs. Most of the data on the association between early microbe exposure and subsequent changes to the immune response come from human epidemiological studies in which the timing, duration, and extent of exposure is often both uncontrolled and undetermined. Additional studies using animal models are required to gain insight into the precise mechanisms by which perinatal inflammation drives lasting changes to offspring immune function. Based on advancements in understanding of both hematopoietic and immune development over the last decade, we posit at least two probable mechanisms by which perinatal inflammation could influence long-term immune outcomes in offspring: first, by perturbing the development and function of fetal-derived immune cells, and second, by perturbing the establishment and function of developing hematopoietic stem cells (Figure 1).

### 1.1. Contribution of Fetal-Derived Immune Cells to Tissue Immunity

The discovery that fetal immune cells contribute to tissue development and immunity is arguably one of the most exciting recent discoveries within the field of immunology. Genetic fate mapping has revealed that many adult tissue-resident immune cells are generated during fetal life, including macrophages [8], innate-like lymphocytes, including B1-a cells [24,25], innate lymphoid cells [10], and γδ-T cells [25], and mast cells [26]. The best example of these are tissue-resident macrophages (TRMs), for which accumulating evidence in both mouse models [8,27] and humans [28] supports a sustained contribution of fetal cells to postnatal immunity. Fetal-derived TRMs are generated in waves from the earliest, “primitive” fetal hematopoietic precursors in the extraembryonic yolk sac, as well as later hematopoietic progenitors in the fetal liver (discussed below). These cells are thought to migrate to and seed developing tissues during the fetal period, where they differentiate and establish residency, and subsequently contribute to tissue development and homeostasis. As they differentiate and seed developing tissues, fetal macrophages acquire distinct functions within their tissues of residence, underscored by the acquisition of unique transcriptional and enhancer landscapes [29,30,31]. Once seeded, fetal-derived TRMs are capable of self-maintenance within their tissues of residence; they are radio-resistant, and are poorly regenerated by adult hematopoiesis [32].

There are several different routes by which perturbed development of fetal-derived immune cells might influence tissue health and function, and thereby drive disease pathogenesis. Recent examples from the literature suggest that fetal-derived TRMs, and likely other fetal-derived immune cells, participate in normal tissue *development*. Embryonic-derived microglia invade the central nervous system before neurogenesis [33], and eliminate apoptotic neurons by phagocytosis and prune synapses as the brain develops [34,35]. Depletion of microglia impairs axonal outgrowth and neuronal connectivity during brain development [11]. Similarly, depletion of macrophages in fetal kidney ex vivo results in loss of vascularization [12]. Fetal-derived macrophages in the endocardium regulate valvular remodeling during cardiac development [13] as well as vascular organization and testis cord morphogenesis during testis development [14]. Although there are currently only a limited number of direct examples from the literature of fetal-derived macrophages regulating organ development, this is an area of active investigation. The intricacies and interactions of both fetal organ development and fetal immune development make investigation of precise developmental functions challenging; the proper model or system with which to dissect the specific contributions of overlapping yet distinct macrophage subsets to tissue development has been difficult to identify. Ultimately, perturbed interactions between developing immune cells and developing tissues may lead to tissues that are not properly formed, yielding them susceptible to dysfunction and disease.

Perturbation of fetal immune development might also ultimately influence tissue health and function by affecting tissue *homeostasis*. If the *function* of fetal-derived tissue-resident cells is altered, this might influence immune homeostasis of different tissues from development onwards. An increasing number of studies provide evidence that fetal-derived tissue-resident macrophages directly regulate tissue homeostasis in the gut [36], heart [37,38], and lungs [39], among other tissues, and may play roles in regulating adaptive responses in the face of injury [40]. Again, as this is a burgeoning field, there are only a few very recent examples of dysregulated fetal immune cells impairing tissue homeostasis postnatally. However, one can envision that perturbed function of cells that maintain homeostasis might reset the immune landscape of developing tissues, thereby setting the stage for immune dysregulation and tissue dysfunction.

Given that adult hematopoiesis is generating and replacing immune cells daily, it is conceivable that fetal-derived immune cells could be replaced under developmental conditions in which their establishment has been perturbed. In studies that have directly examined the capability of adult bone marrow hematopoiesis to replace fetal-derived immune cells, the answer remains ambiguous. Fetal-derived cells *can* be replaced by adult hematopoiesis in some tissues under certain conditions. Using a genetic deletion model, Guilliams and colleagues demonstrated that bone marrow precursors possessed the same capability to seed an empty niche for alveolar macrophages in the lung as compared to yolk sac or fetal liver precursors, and bone marrow-derived alveolar macrophages were transcriptionally and functionally comparable to those derived from fetal precursors [41]. However, whether adult-derived cells are functionally equivalent to fetal-derived cells is of great debate. For example, microglia can be replaced by adult precursors under conditions where they have been conditionally depleted from their niche; however, these cells are not transcriptionally identical [42]. Furthermore, whether adult-derived tissue-resident macrophages are functionally redundant under both homeostatic and disease states remains to be determined. Despite identical phenotypes, subtle functional and transcriptional differences between fetal-derived and adult-derived tissue-resident cells may have important functional consequences for disease susceptibility, as suggested by the distinct contribution of fetal-derived macrophages to tumor-associated macrophages and tumor progression [43]. Future studies will address whether differences between “original” fetal-derived immune cells and adult-derived “replacements” contribute to deterioration of tissue homeostasis or disease pathogenesis.

### 1.2. Susceptibility of Hematopoietic Stem Cell Development to Perinatal Inflammation

Hematopoietic stem cells (HSCs) are responsible for the production of all blood and immune cells across the lifespan. Adult HSCs are maintained within the bone marrow niche, where they are seeded during late gestation in humans and just after birth in mice. Prior to seeding the bone marrow, the establishment of the hematopoietic system is a highly dynamic process that occurs across multiple anatomical sites during development. The first hematopoietic progenitors arise from hemogenic endothelium in the extraembryonic yolk sac, and primarily generate primitive red blood cells to meet the early oxygenation needs of the developing embryo [44]. This earliest “primitive” wave of hematopoiesis is rapidly superseded by overlapping waves of hematopoietic cell production from increasingly mature progenitors. Erythromyeloid progenitors subsequently arise within the yolk sac and developing aorta, and generate increasingly mature erythroid and myeloid progeny, including macrophages and megakaryocytes, as well as the first lymphoid cells in the embryo [45]. The first definitive HSCs capable of generating all mature hematopoietic cell lineages are thought to arise from hemogenic endothelium in the developing aorta, although a yolk sac origin of definitive HSCs has also been suggested [46,47].

Across development, the hematopoietic system has to constantly shift to meet the dynamic needs of the early embryo, which differ starkly from those of the adult. This is very clearly elucidated in the rapid layering of the erythroid system, in which globin switching across development facilitates critical changes in oxygenation capacity [48]. In a similar vein, Herzenberg and colleagues proposed the concept of “layered immune development,” based on the discovery of subsets of functionally-distinct B cells produced only during fetal life [49]. At a very basic level, layered immunity explains the shift from the production of tolerogenic, innate-like immune cells early in life that facilitate maternal-fetal tolerance but also support a first line of protection against pathogens, to the production of a mature adaptive immune system by adult hematopoiesis later in development. Although the concept of layered immunity is several decades old, the hypothesis has recently gained more traction as an increasing number of cell types, including innate lymphoid cells [10], CD8 T cells [50], mast cells [26], and others have been shown to be functionally layered across ontogeny.

We and others have demonstrated that layered immunity is accomplished via the production of distinct immune cells from transient progenitors with distinct potential [25,51,52]. Indeed, in vivo lineage tracing has supported the concept that many fetal progenitors are transient, disappearing before adulthood with limited contribution to adult hematopoiesis [51,52,53]. Under homeostatic conditions, the limited contribution of such transient progenitors may serve to precisely “layer” the immune system such that tolerogenic cells produced in early life are produced in limited number and capacity, to be replaced by adult-derived cells of greater maturity and differing function. In contrast, perinatal inflammation may alter the landscape of layered immunity, both at the progenitor level and at the level of mature cell output. For example, perinatal inflammation could cause the loss of transient early progenitors and the subsequent loss of early immune cells important for early life tolerance. Conversely, perinatal inflammation could feasibly drive the inappropriate expansion and persistence of otherwise transient progenitors, causing expansion and continued production of fetal- immune cells that would otherwise be restricted to a specific developmental window. Perinatal inflammation during hematopoietic development could also impinge upon the establishment of adult HSC precursors, resulting in changes to HSC composition, quiescence, metabolism, and response to stress (Figure 1).

None of these possibilities have been directly investigated to date. However, clues from studies of the adult HSC response to inflammation shed some light on how fetal HSCs might respond to perinatal inflammation. Adult HSCs act as sensors of inflammation, responding directly to a range of inflammatory cues, including cytokines, Toll-like receptor agonists, and immunomodulators [54]. Acute inflammation activates HSCs, causing them to exit quiescence and rapidly proliferate to restore immune homeostasis. Both acute and chronic inflammation also invoke a rapid myeloid-biased response, potentially by activating myeloid-biased HSCs [55], and provoking downstream expansion of myeloid-biased progenitors [56,57]. These responses necessarily reflect the composition of the adult HSC compartment—whether the same outcomes are true of the fetal HSC compartment remain to be determined. Despite being poorly characterized as compared to the adult HSC compartment, we know that the fetal HSC compartment differs fundamentally from the adult HSC compartment. The fetal HSC compartment contains more lymphoid-biased progenitors [58], are less quiescent [59,60], express different phenotypic markers suggestive of functional differences [61,62], and produce distinct mature cell output. These underlying differences suggest that the response of fetal hematopoiesis to perinatal inflammation might be fundamentally different than the adult response. Additionally, whereas adult HSCs in the bone marrow are exposed directly to inflammatory mediators via the bloodstream, fetal HSCs are “protected” from inflammation via the maternal-fetal barrier. There is very little information regarding how the fetus senses and interprets maternal infection and inflammation, and how this is perceived and responded to by developing fetal HSCs. Future work will need to address these knowledge gaps.

## 2. The Cochlea as a Model Organ to Study the Effect of Early Life Inflammation on the Developing Hematopoietic and Immune Systems

A model system is needed to assess how the effects of perinatal inflammation converge on immune development, hematopoietic development, and tissue development. Here, we propose the auditory system, specifically the cochlea, as an excellent candidate for modeling how disruptions to perinatal hematopoietic and immune development impinge upon tissue development and homeostasis to cause disease. The remainder of this review will present several arguments to support investigation of fetal immune and hematopoietic development in the context of the cochlea. First, we will describe the cochlea as a sensitive organ to a wide range of insults, with an emphasis on congenital infection as an important tool in understanding how early inflammation disrupts perinatal immune development. Next, we will explore the unique structural and functional characteristics of the cochlea that allow for accurate and targeted measurement of impairment in response to injury or inflammation. Finally, we will discuss the growing body of knowledge examining the developing and mature cochlear immune milieu and the role of the developing immune system in mediating cochlear dysfunction.

### 2.1. The Cochlea Is Sensitive to a Broad Range of Inputs

Sensorineural hearing loss (SNHL) is highly prevalent, affecting approximately 50% of American adults over 65 years of age [63], and 2 to 3 of every 1000 infants at birth [64,65]. Congenital infection is an important cause of SNHL and many different congenital infections can cause SNHL including toxoplasmosis, rubella, syphilis, zika, HIV, and cytomegalovirus (CMV) [66,67]. Congenital cytomegalovirus (cCMV) is the most common congenital infection worldwide, and is the number one cause of non-genetic SNHL and a leading cause of central nervous system defects in newborns [68,69,70]. The number of children with cCMV-related sequelae is similar to or greater than the number with better known conditions such as Down syndrome or spina bifida [71]. Hearing loss has detrimental effects on speech and language development and incurs the major cost associated with cCMV infection, which has been estimated to be $4 billion a year [72]. Considering that not just one, but many congenital infections are significant causes of SNHL, it is evident that the cochlea is an extremely developmentally sensitive organ to early immune perturbation.

Examination of the timing, duration, and severity of exposure during congenital infection on SNHL suggests that prenatal inflammation is contributing to cochlear damage in SNHL. For example, mere exposure to HIV during prenatal development causes hearing loss in children, though infection is associated with higher prevalence of hearing loss as compared perinatal exposure only [73]. In the case of rubella virus and CMV infection, exposure during the first trimester causes worse and more frequent hearing loss [74,75]. Furthermore, for both CMV and rubella virus infections, infants presenting with systemic, symptomatic infection reflecting disseminated infection are more likely to progress to severe or profound hearing loss [76,77]. Notably, CMV and rubella virus infection acquired postnatally have not been found to cause hearing loss [78,79], suggesting that prenatal cochlear development is particularly sensitive. Importantly, different congenital infections also appear to cause hearing loss via disparate mechanisms. Whereas hearing does not progressively worsen in rubella-infected infants, progressive hearing loss is a hallmark of CMV infection, highlighting a likely difference between the mechanism of these two infections [75,80,81,82]. Cytopathic effects have been observed in cochlear structures of CMV- and rubella-mediated SNHL, though the mechanism of this damage is poorly understood [83].

Although antimicrobial treatments have been shown to improve hearing outcomes for some infections such as toxoplasmosis and syphilis [84,85,86], there is no current effective treatment or vaccine for congenital CMV [87,88]. Several groups have proposed that persistent inflammation may be responsible for the worsening of hearing in CMV-infected infants [89,90,91]. As SNHL in response to CMV infection only occurs in the context of congenital infection—postnatal infection does not cause SNHL—the response of the fetal immune system is implicated in initiating persistent inflammation. Indeed, the fetal immune system is capable of mounting a robust immune response to CMV [92]. The specific role of this response in driving human pathogenesis in response to congenital CMV still remains unclear, but work in mouse models suggests that this early immune response drives clinical manifestation in neuropathogenesis [93].

Based on the susceptibility of the cochlea to a wide array of congenital infections, we view the cochlea as an ideal system to examine how perinatal inflammation drives persistent tissue dysfunction by impairing immune development. Given the enormous public health impact of cCMV on childhood hearing, we focus specifically on congenital CMV infection as a model to elaborate the cochlea’s utility for the study of inflammation on hematopoietic and immune development.

### 2.2. Hearing Testing Accurately Assesses Cochlear Damage

An important attribute of the cochlea as a model system is that the hearing phenotype often accurately reflects its function [94]. The auditory system is organized in a manner that permits accurate and precise assessment of cochlear function using hearing phenotype [94]. To illustrate this point, we will briefly review the structure and function of the auditory system and cochlea, and the hearing testing methods utilized to assess cochlear function.

**Figure 2:**
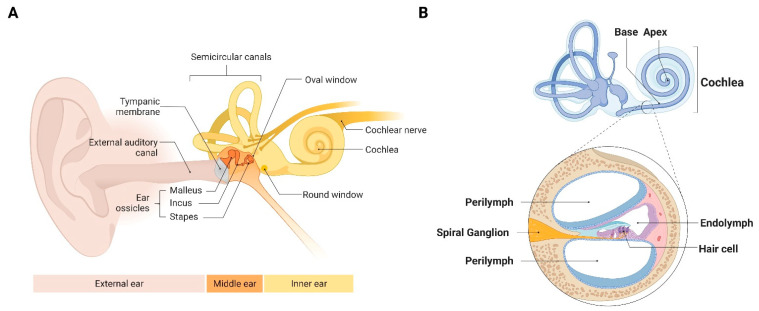
Anatomy of the ear and auditory system. (**A**). Outer, middle, and inner ear shown with labelled anatomic structures that are important for hearing. (**B**). Illustration of a human cochlea with the cochlear base and apex labelled. Hair cells at the cochlear base interpret high-pitch sounds and hair cells at the apex interpret low-pitch sounds. The cross section of the cochlea shows the location of perilymph and endolymph. Hair cells resonate with incoming sound waves and send impulses to the brain via the spiral ganglion.

As sound waves enter the ear canal, they are amplified via vibration of the ear drum and a small chain of bones in the middle ear, called the ossicles, which then transmit through the oval window to the cochlea (Figure 2A). As the oval window oscillates, it sends waves through the fluid within the cochlea called the perilymph. These fluid waves displace the stereocilia located on the apical end of the inner hair cells (IHC) which are specialized cells surrounded by potassium-rich endolymph. A blood labyrinthine barrier (BLB) is created between the perilymph and endolymph fluids which is essential for normal inner ear function. The endolymph, the fluid within the inner ear membranous labyrinth and the perilymph, the fluid that surrounds this membranous labyrinth differ markedly in their potassium composition [95]. This potassium ion gradient creates an endocochlear potential which is maintained by a network of blood vessels in the lateral wall of the cochlea called the stria vascularis (Figure 2B). The topographic arrangement of the cells enables specific hair cells to depolarize in a frequency-specific fashion, resulting in opening of channels to mediate entry of potassium ions that generate an action potential. These coordinated steps successfully convert mechanical sound waves into nerve impulses that are transmitted to the spiral ganglion of the cochlear nerve, and then travel along the cochlear nerve to the brain for interpretation. A second type of mechanosensory cell is the outer hair cell (OHC). This cell also transduces the mechanical force generated by sound waves into an electrical signal. OHCs are responsible for an active mechanical amplification process that leads to fine tuning and increased sensitivity to sound inputs. Dysfunction of any of these structures can lead to hearing loss, although OHCs tend to be the most vulnerable to injury.

**Figure 3:**
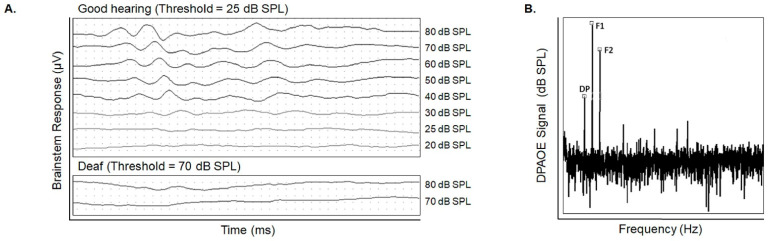
Sensorineural hearing loss can be assessed by auditory brainstem response (ABR) and distortion product otoacoustic emissions (DPOAE) testing. (**A**). Comparison of ABR waveforms from a mouse with normal hearing and a mouse with severe to profound hearing loss. Higher thresholds indicate worse hearing, and lower thresholds indicate better hearing. Detected ABR signals are measured in microvolts (μV) over time in milliseconds (ms), and hearing thresholds are determined as decibels of sound pressure level (dB SPL) based on the presented auditory stimulus. (**B**). DPOAE results showing peaks from presented stimuli (Frequency 1 [F1] and Frequency 2 [F2] peaks) and the presence of a distortion product (DP peak). The presence of the distortion product indicates that OHCs are responding properly to the noise stimulus with detected signals measured in dB SPL.

A key characteristic of the cochlea is its topographical organization. Hair cells located near the base of the cochlea respond to high-frequency sound waves, and hair cells near the cochlear apex respond to low-frequency sound waves (Figure 2B). We can perform frequency-specific measurements of auditory function through established hearing assessment methods using distortion product otoacoustic emission (DPOAE), auditory brainstem response (ABR) and behavioral testing (in humans). In ABR testing, probes are placed on the individual’s or animal’s head to measure evoked potentials milliseconds following a sound stimulus. The probes detect responses from the auditory pathway from the cochlea to the brainstem (Figure 3A). DPOAE detect emissions generated from OHCs. In DPOAE testing, two sound frequencies are presented into the ear canal. If functioning OHCs are present, they will resonate with the sound waves and emit a distortion product of the two sound waves (Figure 3B) [96]. Put simply, DPOAE is a more specific measure of functioning OHCs, and ABR is an overall measure of the auditory pathway [95]. Visual reinforced audiometry (VRA) and conditioned play audiometry (CPA) are two other behavioral diagnostic methods of choice in children [97,98]. A key outcome of DPOAE, behavioral and ABR testing is the hearing threshold, or the lowest intensity that elicits a response at a given frequency. Therefore, higher thresholds indicate worse hearing, and lower thresholds indicate better hearing. All methods are performed at high, middle, and low frequencies to measure the cochlea’s sensitivity along the basal, mid, and apical turns, respectively. Combined results of DPOAE, behavioral and ABR testing may indicate whether OHC damage or other cochlear structures mediate SNHL [94]. One exception to the hearing measures being an accurate reflection to function is synaptopathy; clinical audiometric approaches cannot reveal the presence of neurodegeneration in humans [99]). However, cochlear functional assays and confocal microscopy have been used to demonstrate transient thresholds elevation and delayed cochlear nerve degeneration in mice [100].

We have reliably demonstrated that early CMV infection in our mouse model causes elevated thresholds in both ABR and DPOAE testing [101,102]. Specifically, we observed high-frequency hearing loss which worsened over time [103]. Low- and mid-frequency hearing is generally preserved in early hearing testing of infected mice, though hearing at these frequencies also worsens over time. Additional studies from our group have shown extensive degeneration of the stria vascularis, spiral ganglion apoptosis, and loss of endocochlear potential in CMV-infected mice [103,104]. We have also demonstrated mCMV infection results in a synaptopathy before hair cell damage [102]. Together, these data confirm that human cochlear pathogenesis can be recapitulated in a mouse model of CMV infection.

### 2.3. The Cochlea Contains a Rich, Unexplored Immune Milieu

Although the cochlea may be described as a dynamic and immunogenic environment that is susceptible to a wide array of immune insults and pathogens, it was long considered as “immune privileged” due to the presence of tight junctions within the blood-labyrinth barrier (BLB), which prevent entry and establishment of immune cells in the cochlea [105]. Entry of cells into the inner ear by the BLB has also heavily restricted advancement of drug and cell-based therapies for cochlea treatment [106,107]. Perhaps because of this barrier, much work to date on defining the pathology of SNHL has focused on the cochlear architecture, including mechanosensory hair cells, the stria vascularis, the spiral ganglion, or cochlear synapse connectivity [67,102]. More recent work, however, has clarified that a variety of CD45+ immune cells are present in the mouse cochlea under normal physiological conditions [90,108,109,110,111] and seed cochlear tissue during fetal and neonatal development, before the formation and maturity of the BLB [112,113,114]. Work defining early cochlear immunity is still in its early stages; two very recent studies [110,113] have examined heterogeneity of mature immune populations in postnatal and adult murine cochlea. In the postnatal day 4 cochlea, CD11b+Gr1- macrophages comprised 80% of cochlear immune cells, with minimal contribution of NK cells, granulocytes, B and T cells [113]. In adult mouse cochlea, CD11b+ myeloid cells are the most abundant cell types, of which CX3CR1+ macrophages comprise 30% of CD11b+ cells. B cells, NK cells, T cells, and neutrophils are also present in adult cochlea at higher frequencies as compared to postnatal day (PD) 4, indicating that the composition of immune cells changes across postnatal development within the immature cochlea. Additional characterization is clearly required to define the immune composition of the cochlea across ontogeny.

The role of the developing BLB in regulating cochlear immune development remains to be determined. A handful of studies have reported that the BLB matures postnatally in both the rat and in the mouse. Administration of polyethylenemine (PEI) systemically to determine its distribution on the cochlear capillary basal lamina of the stria vascularis revealed significantly greater presence of PEI in the stria vascularis of 4-, 7- and 11-day-old rats as compared to that of adult rats [115]. Similarly, GTTR uptake was elevated in the hair cells of 6-day-old mice or when the BLB was compromised by ethacrynic acid as compared to that of 21–28 day-old mice [116]. To our knowledge, there are no studies to date that have evaluated the maturation of the BLB in humans nor the effect of BLB disruption on host immune development.

Although multiple cochlear immune populations have been observed, limited studies to date have focused on the distribution and development of macrophages within the cochlea. Several studies have shown that macrophages are the most abundant immune cell population in cochlea [114,117,118], identified variably by their expression of F4/80 [116,117], Iba1 [114,117,119], CX3CR1 [114,115,119], CD68 [114,116,117,119] and CD11b [115,116,118]. These macrophages are present along the spiral ganglion, spiral ligament, spiral lamina, and stria vascularis throughout postnatal and adult cochlea [91,113,114,117]. Preliminary investigation of the developmental distribution and origin of these macrophages has suggested a fetal origin. Macrophages have been identified as early as E10.5 in the mesenchyme surrounding the otocyst during fetal development [114]. Histological analyses indicated that fetal macrophages seed, expand, and contract in specific spatial niches of the cochlea across fetal development [114]. In the spiral ganglion, macrophages were present prenatally but significantly increased after birth and continued to increase through adulthood. Macrophage numbers in the spiral ligament peaked at postnatal day 3 and continually decreased, whereas macrophages in the stria vascularis were observed to be seeded and expanded after birth, and were therefore only observed postnatally. Thus, the cochlea appears to be seeded during development by macrophages from different sources in a highly spatiotemporally regulated pattern.

The specific role of fetal-derived macrophages in cochlear development was recently investigated by a single global inactivation mutation model in CSF1^op/op^ mice [117] and a deletion model of CSF1R [114], critical regulators of fetal macrophage development. CSF1 inactivation simultaneously impaired cochlear macrophage development and hearing function [117]. However, CSF1^op/op^ mice also develop osteopetrosis, or thickened and dense bone formation [117], and the authors reported narrowing of the endolymph-containing semicircular canals and excessive bone thickening of the otic capsule [117]. Cochlear tissue in CSF1^op/op^ mice also appeared visually normal [117], and in the absence of additional analysis of hair cells, spiral ganglion, and stria vascularis, as well as DPOAE testing, the precise pathogenic mechanisms mediating hearing loss in CSF1^op/op^ mice were unclear. In both CSF1-inactivation and CSF1R deletion studies, cochlea macrophages were significantly depleted in the stria vascularis and spiral ligament in fetal and adult cochlea [114,117]. CSF1-independent macrophages were still found in adult spiral ganglion, but were not sufficient to support healthy cochlear development [117]. While these findings provide the first line of evidence that fetal-derived tissue-resident macrophages are important for cochlear development and hearing function, the specific contribution of distinct fetal macrophage subsets to cochlear development, function, homeostasis, and response to perturbation requires further investigation.

Interestingly, although macrophages are the most studied tissue-resident immune cell, they may not be the only CD45+ immune cells that seed and reside in cochlear tissue. Preliminary examination of mouse cochlear tissue that has been perfused to remove circulating blood and immune cells suggests the presence of other tissue-resident cells, including tissue-resident B cells, T cells, NK cells, and neutrophils [110]. While these cell types are not considered as classical tissue-resident cells, emerging research suggests that these cell types exist as tissue-resident subsets at steady state in other tissues [118,119,120,121,122]. Tissue-resident NK cells have recently been described as functionally immature and undergo maturation in tissue [123] and may play homeostatic roles similar to that of tissue-resident macrophages. Tissue-resident memory B and T cells are associated with long-term protective immunity [124,125,126]. Tissue-resident neutrophils are not well-characterized; however, homeostatic and tissue surveillance roles have been suggested [127]. Minimal evidence suggests the presence of these tissue-resident subsets in other tissues, but they have not yet been defined within the cochlea.

### 2.4. The Developing Cochlea Contains Both Immune and Hematopoietic Stem and Progenitor Cells

**Figure 4:**
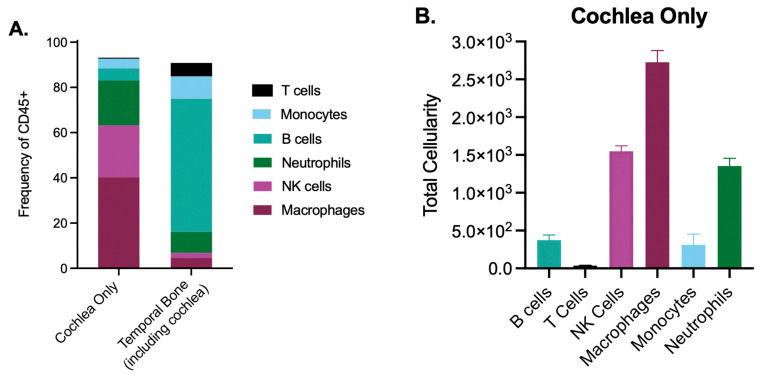
The developing cochlea contains a rich immune environment. Flow cytometric analysis of cochlear and temporal bone immune populations of post-natal day 10 mice. Cochlea and temporal bone were dissected as described in the methods section and similar to Jan et al. [128]. (**A**). Comparison of innate and adaptive immune populations as defined in the methods section as a percentage of CD45+ cells. (**B**). Total cellularity of innate and adaptive immune populations in the cochlea. Error bars represent the mean ± SEM. N = 5–6 mice per tissue structure.

Studies investigating SNHL and associated cochlear damage have not delineated between the structure of the cochlea and the surrounding temporal bone, and have used these terms interchangeably. The temporal bone includes the inner ear structures of the cochlea as well as the vestibular system. As the temporal bone contains cells of non-cochlear origin, including the surrounding bone marrow, it may be important to analyze the cochlea and the temporal bone separately in the context of congenital infection, particularly by flow cytometric analysis. Recent studies [110,113] have begun to characterize the cochlea specifically by dissecting the cochlea from the temporal bone using an optimized protocol described by Jan and colleagues [128]. In this protocol, cochlear capsule is chipped away and cochlear tissue is removed from surrounding bone and otic capsule. We recently adapted this protocol to compare the immune profile in the cochlea at postnatal day (PD)10 versus the whole temporal bone including the cochlea (Figure 4A). We observed vast differences in the immune composition between these two structures.

Striking differences were observed when the temporal bone was included in analysis of cochlear immune populations. Overall, our analysis at PD10 revealed the identity of 93.1% of live CD45+ cochlear immune cells, and 90.8% of live CD45+ temporal bone immune cells. In the cochlea only, live CD45+ cells were composed primarily of macrophages (40.3%, F4/80^hi^CD11b+), followed by NK cells (22.9%, NK1.1+), and neutrophils (19.9%, Ly6G+). Only a small fraction of cochlear cells included B cells (5.3% CD19+), monocytes (4.2%, F4/80^mid^CD11b+), and T cells (0.5%, CD3+). In contrast, the temporal bone was composed primarily of B cells (58.8%), with smaller fractions of monocytes (10.0%), neutrophils (9.4%), T cells (5.9%), macrophages (4.6%), and NK cells (2.2%). The same surface markers were used to identify immune populations within the temporal bone and cochlea. Overall, immune cell frequencies in the PD10 cochlea were similar to those reported in the adult cochlea [110]. Notably, our findings suggest a greater number of identified immune cells (Figure 4B) than previously published data, as well as characterize a higher frequency of CD45+ cochlear immune cells. However, surface markers used to identify immune populations often differ across different studies, particularly for myeloid populations which can be alternately defined using markers such ascF4/80, Gr-1/Ly6G, Ly6C, CD11c, CX3CR1, and Iba1. Varying combinations of these markers to identify myeloid populations may account for reported differences in frequencies. More refined protocols will be important for ascertaining cochlear-specific immune populations and defining homeostatic immune populations at baseline in comparison to infiltrating immune populations present during inflammatory conditions.

**Figure 5:**
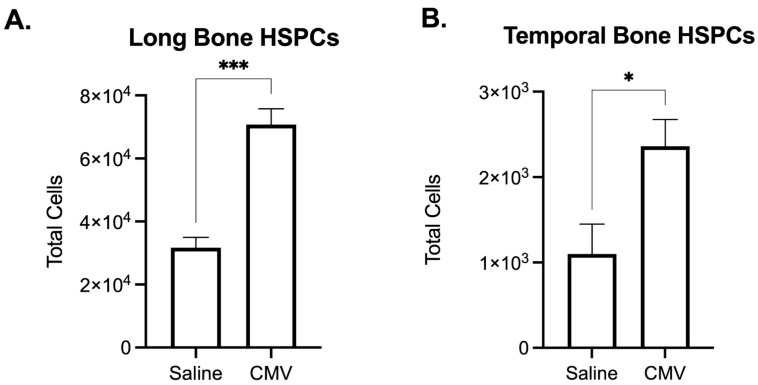
Temporal bone hematopoietic stem and progenitor cells (HSPCs) are sensitive to perinatal infection with CMV. Flow cytometric analysis of HSPCs (Lin− c-kit+ Sca-1+) of post-natal day 10 mice that were treated with saline or mCMV at post-natal day 3. (**A**). Long bone (femur and tibia) marrow HSPCs significantly expand in response CMV infection (7dpi). (**B**). Temporal bone contains fewer HSPCs than tibial bone marrow, but similarly expand in response to CMV infection. Error bars represent the mean ± SEM. N = 7–13 mice per treatment across 2–4 litters. * *p* < 0.05, *** *p* < 0.001.

Given that the temporal bone also contains bone marrow, we investigated whether we could analyze bone marrow hematopoietic stem and progenitor cell (HSPC) populations within the temporal bone, to simultaneously investigate the effects of congenital infection on both developing HSPCs within bone marrow as well as developing immune cells within the cochlea. As proof of principle, we infected mice with mCMV at PD3 and compared the effects of CMV infection on HSPC cellularity in the temporal bone and long bone (femur and tibia) marrow. Although significantly fewer HSPCs were detected in the temporal bone as compared to the long bone, both HSPC populations were significantly expanded in response to CMV infection (Figure 5). These preliminary data suggest that changes within temporal bone HSPC populations may be used as a proxy to define perturbation to developing bone marrow HSPCs.

### 2.5. Persistent Inflammation May Contribute to SNHL

A core feature of pediatric CMV-induced SNHL is that it becomes progressively worse with time [75]. In our mouse model, we have observed that CMV infection has an initial acute phase lasting one week, with active replication of the virus followed by an indefinite phase without observed active replication [129]. Interestingly, hearing continues to worsen weeks after active infection [103]. This progressive worsening of hearing in the context of non-active infection has driven us to question whether direct viral cytopathogenic effects, or other factors, are contributing to progressive hearing loss. However, one observation is clear: early perturbation to auditory system homeostasis has both immediate and persistent effects on hearing function.

We postulate that early CMV infection causes an early and persistent inflammatory state in the cochlea which drives the development of progressive SNHL. The hypothesis that early immune dysregulation is a driver for CMV-induced SNHL is supported by a limited yet growing body of evidence. For example, a persistent macrophage population was identified in the cochlea of CMV infected mice and hypothesized to be a source of reactive oxygen species (ROS) production [90]. CMV infection also induced an inflammatory reaction in the cochlea for which treatment with steroids simultaneously improved hearing outcomes, reduced inflammation, and prevented damage to cochlear structures [130]. In the same study, the degree of inflammation, and not viral load, was correlated with hearing outcomes. Our lab has also demonstrated that host-derived ROS played a role in CMV-mediated SNHL, and that treatment with anti-oxidants reduced ROS, thereby improving hearing outcomes [131]. Viral latency and evidence of inflammation has also been observed within the human cochlea following CMV infection [132,133]. Together, these data suggest that in the absence of active viral infection, sustained inflammation from early life onwards contributes to degradation of cochlear function and SNHL.

## 3. Conclusions: Perturbation of Fetal Immunity as a Driver of SNHL

Perinatal inflammation has the propensity to affect long-term health outcomes and disease susceptibility by perturbing both the developing hematopoietic and immune systems from multiple angles. Disrupting the establishment of tissue-resident cells that seed and persist in developing tissues during early life could compromise long-term tissue development, function, and homeostasis. As hematopoietic stem cells are sensitive to inflammation [54], perinatal inflammation could have a potent impact on the development, function, and composition of developing hematopoietic stem cells, and thereby alter the trajectory of immune output.

Here, we propose that the developing cochlea is an ideal system to directly examine how perinatal infection and inflammation drives disease pathogenesis. We established an association between congenital infection and progressive cochlear dysfunction that results in sensorineural hearing loss (SNHL). The cochlea is an exquisitely delicate structure, and frequency specific hearing thresholds are particularly sensitive measures of tissue damage, since tissue dysfunction can be directly mapped to tissue architecture. These features allow more precise detection and quantification of the effects of inflammation—including timing, duration, and degree of exposure—on tissue development and function. The cochlea is susceptible to prenatal inflammation, and we summarize accumulating evidence that the cochlea contains a rich immune milieu that is populated during the perinatal period, and may regulate hearing function [117]. Indeed, perinatal treatment with anti-inflammatories and anti-oxidants prevents SNHL induced by congenital infection in mouse models, providing initial evidence that inflammation is a driver of progressive hearing loss. Lastly, as an anatomical structure, the cochlea contains both the hearing organ as well as temporal bone. As we demonstrate, both bone marrow hematopoietic progenitor populations and cochlear immune populations can be analyzed simultaneously to define the effects of congenital infection and perinatal inflammation on the developing immune and hematopoietic systems. This last point is extremely powerful: defining and dissociating the effects of perinatal inflammation on developing immune cells and hematopoietic stem cells is crucial for delineating how early life perturbations drive lasting alterations to health and disease susceptibility.

## 4. Materials and Methods

### 4.1. Mice

All mice maintained in the University of Utah vivarium according to IACUC-approved protocols. Mice on C57BL/6 background were used for all experiments.

### 4.2. Viruses

Recombinant mCMV (strain k181 MC.55 [ie2-GFP+]) was provided by Dr. Mark R. Scheiss (Minneapolis, MN). As previously described, virus was propagated in mouse fibroblast cells then purified using centrifugation in a sucrose cushion [103,134]. Stock titers were prepared in murine fibroblast cells and stored at −80 °C.

### 4.3. Viral Inoculation

Mice were inoculated with 1 µL mCMV (200 pfu/µL) on postnatal day 3 via intracerebral injection as previously described [134]. Briefly, mouse pups were anesthetized by being temporarily placed on ice prior to injection. They were then manually restrained as viral inoculum was delivered to the right cerebral hemisphere using a Hamilton syringe with 30G needle. Control animals were similarly injected with 1 µL 1X PBS (−/−).

### 4.4. Cochlea Tissue Processing

Samples were prepared following a previously published procedure for purifying immune cells from the mouse cochlea [128]. The following modifications were used: microdissection of cochlea from individual 10-day-old mice were pooled and harvested into ice cold 5 mM EDTA in 1X PBS (−/−) with 2% BCS. Cochlea were digested in TrypLE Express (Gibco) for 30 min at 37 °C, gently triturating every 15 min to dissociate cells. Cochlea were filtered through a 70 μm filter on top a 5 mL FACS tube. To stop digestion, the filter was rinsed multiple times with 1X PBS (−/−) with 0.5 mM EDTA and 2% BCS. Cochlear cells were centrifuged at 400× *g* for 5 min.

### 4.5. Temporal Bone Processing

Samples were prepared as previously described [90]. The following modifications were used. Individual temporal bones from 10-day-old mice were harvested in ice cold 1X PBS (−/−). Temporal bones were mashed with a blunt end of a syringe and digested in TrypLE Express (Gibco) for 30 min at 37 °C. Temporal bone were filtered through a 70 μm filter on top a 5 mL FACS tube. To stop digestion, the filter was rinsed multiple times with 5 mM EDTA in 1X PBS (−/−) with 2% BCS. Temporal bone cells were centrifuged at 400× *g* for 5 min.

### 4.6. Long Bone Processing

Bone marrow was isolated by flushing both femurs and tibias from each mouse with 5 mM EDTA in 1X PBS (−/−) with 2% BCS. Bone chips were filtered out using a 70 μm filter on top a 5 mL FACS tube. Red blood cells were lysed using 1X ACK Lysis buffer and then resuspended in 5 mM EDTA in 1X PBS (−/−) with 2% BCS. Long bone marrow cells were centrifuged at 400× *g* for 5 min.

### 4.7. Flow Cytometry

Surface staining and secondary staining were performed in 5 mM EDTA in 1X PBS (−/−) with 2% fetal calf serum for 30 min at 4 °C. For comparison of immune populations between cochlea and temporal bone, samples for were stained with the following antibodies: anti-mouse CD45 (104), anti-mouse CD3 (145-2C11), anti-mouse CD19 (6D5), anti-mouse Ly6G (1A8), anti-mouse NK1.1 (PK136), anti-mouse CD11b (M1/70), and anti-mouse F4/80 (BM8). Hematopoietic stem and progenitor populations in long bone and temporal bone samples were stained with the following antibodies: lineage markers [anti-mouse CD3 (145-2C11), anti-mouse CD4 (GK1.5), anti-mouse CD8 (53-6.7), anti-mouse CD5 (53-7.3), anti-mouse CD19 (6D5), anti-mouse NK1.1 (PK136), anti-mouse Ter-119 (TER-119), anti-mouse Gr-1 (RB6-8C5), anti-mouse F4/80 (BM8)], anti-mouse CD45 (104), anti-mouse c-kit (2B8), anti-mouse Sca1 (E13-161.7), and Streptavidin. DAPI or PI was used to preclude dead cells. Data were acquired on Aurora spectral analyzer (Cytek) and analyzed using FlowJo (BD) and Prism (Graphpad) software.

### 4.8. Gating Strategies for Flow Cytometry

Immune cell and progenitor populations were gated as viable singlets, and further defined as follows:

T cells: CD45+ CD3+;

Monocytes: CD45+ F4/80mid CD11b+;

B cells: CD45+ CD19+;

Neutrophils: CD45+ Ly6G+;

NK cells: CD45+ NK1.1+;

Macrophages: CD45+ F4/80+ CD11b+;

HSPCs: CD45+ Lin- ckit+ Sca1+.

### 4.9. Statistical Analysis

Graphs, average values, and standard error mean (SEM) shown in figures were calculated using Prism (version 9.1.0 Graphpad) software. The effects of CMV were analyzed by non-parametric Mann–Whitney *U* test. Number of n and *p*-values less than 0.05 are described in each figure legend.

## Data Availability

The data presented in this study are available on request from the corresponding author.
